# Identification and molecular detection of the pathogen of *Phalaenopsis* leaf yellowing through genome analysis

**DOI:** 10.3389/fmicb.2024.1431813

**Published:** 2024-09-24

**Authors:** Wei-Chin Tsao, Yi-Hsuan Li, Yi-He Tu, Yu-Shin Nai, Tsung-Chun Lin, Chih-Li Wang

**Affiliations:** ^1^Department of Plant Pathology, National Chung Hsing University, Taichung, Taiwan; ^2^Doctoral Program in Microbial Genomics, National Chung Hsing University and Academia Sinica, Taichung, Taiwan; ^3^Department of Entomology, National Chung Hsing University, Taichung, Taiwan; ^4^Plant Pathology Division, Taiwan Agricultural Research Institute, Ministry of Agriculture, Taichung, Taiwan; ^5^Master Program in Plant Medicine and Good Agricultural Practice, National Chung Hsing University, Taichung, Taiwan; ^6^Smart Sustainable New Agriculture Research Center (SMARTer), National Chung Hsing University, Taichung, Taiwan

**Keywords:** moth orchid, *Fusarium phalaenopsidis*, *Fusarium solani* f. sp. *phalaenopsis*, phylogeny, comparative genomics, virulence gene, primer

## Abstract

Moth orchids (*Phalaenopsis* spp.) are globally popular ornamental flowers. However, effective management strategies for *Phalaenopsis* leaf yellowing remain elusive, making the disease a challenging obstacle affecting moth orchids at various growth stages. This disease manifests as collar rot, leaf yellowing, leaf abscission, and eventually, plant death. The lack of effective management strategies is likely attributed to a limited understanding of the disease pathogenesis and pathogen dissemination pathways. *Fusarium phalaenopsidis* sp. nov. was established in this study to stabilize the classification status of *Phalaenopsis* leaf yellowing pathogens using molecular and morphological features. The genome of the holotype strain was sequenced and assembled, revealing its genome structures. Analyses of virulence-related elements, including transposon elements, secondary metabolite biosynthetic gene clusters, effectors, and secreted carbohydrate-active enzymes, shed light on the potential roles of three fast core chromosomes in virulence. Two species-specific primers were designed based on unique gene sequences of two virulence-related proteins through comparative genomics and BLAST screening. The specificity of these primers was validated using isolates of *F. phalaenopsidis*, non-target species in the *Fusarium solani* species complex, other *Fusarium* species complexes, and saprophytic fungi. These results are intended to accelerate the identification of the pathogens, facilitate the study of disease pathogenesis, and pave the way for elucidating pathogen dissemination pathways. Ultimately, they aim to contribute to the formulation of effective control strategies against *Phalaenopsis* leaf yellowing.

## 1 Introduction

Moth orchids (*Phalaenopsis* spp.) are globally recognized as popular ornamental flowers and significant economy elements in Taiwan. The exported value from moth orchids accounts for approximately 70% of the total value of exported flowers. Leaf yellowing disease of *Phalaenopsis* is a significant challenge to the orchid industry, manifesting at various growth stages (Chung et al., 2011; Liao et al., 2012). Despite intensive fungicide treatments, the disease still emerges, especially under the stresses during ocean freight shipping, leading to considerable economic losses (Liao et al., 2012). The disease often occurs with incidence rates frequently ranging between 30 and 60% in susceptible orchid varieties (Su et al., 2010). Affected orchids exhibit necrotic rot at the leaf collar, resulting in leaf chlorosis, leaf abscission, and ultimately plant death.

*Phalaenopsis* leaf yellowing is attributed to *Fusarium solani* f. sp. *phalaenopsis*. Pathogenicity assays have shown that this pathogen can also affect *Cymbidium* sp. with mild symptoms but does not impact *Oncidium* sp., *Dendrobium* sp., *Cattleya* sp., *Pisum sativum*, *Chrysanthemum indicum*, and *Cucumis melo* (Chung et al., 2011). *Fusarium solani*, based on the morphological species concept, is now recognized as the *Fusarium solani* species complex (FSSC), encompassing multiple phylogenetic clades (Schroers et al., 2016). To enhance taxonomic clarity within the FSSC, Schroers et al. (2016) epitypified the type strain of *F. solani sensu stricto* within FSSC 5. Subsequently, several new species within the FSSC have been established, including *F. stercicola*, *F. witzenhausenense* (Šišić et al., 2018a), *F. euwallaceae* (Freeman et al., 2013), *F. cucurbiticola*, *F. petroliphilum*, and *F. phaseoli* (Geiser et al., 2021). Given that the phylogenetic relationships between these species and *F. solani* f. sp. *phalaenopsis* remain unexplored, it is essential to determine the phylogenetic position of *F. solani* f. sp. *phalaenopsis* within the FSSC and establish its taxonomic classification.

Although leaf yellowing disease poses a significant challenge to *Phalaenopsis* spp. production, effective disease management strategies remain elusive (Liao et al., 2012). This gap in disease management is due to the limited understanding of disease pathogenesis and pathogen dissemination. Notably, despite the economic importance of *Fusarium* species within the *Fusarium solani* species complex (FSSC) as agricultural pathogens, research on their virulence factors to plant hosts is sparse (Coleman, 2016). Advancements in whole-genome sequencing technology have uncovered genomes of key FSSC pathogens, including *F. vanettenii* (Coleman et al., 2009), *F. virguliforme* associated with soybean SDS (Srivastava et al., 2014), species in ambrosia *Fusarium* clade (AFC) (Short et al., 2017), and *F. solani-melongenae* (Xie et al., 2022). This genomic information paves the way for comprehensive molecular research on these pathogens. Therefore, establishing a genome database for the pathogen of *Phalaenopsis* leaf yellowing is imperative.

Comparative genomics has been utilized to construct genome structures and identify unique genes within a species (Rep and Kistler, 2010; Ma et al., 2013). *Fusarium vanettenii* 77-13-4 (formerly known as *N. haematococca* mpVI 77-13-4) was the first species within the *Fusarium solani* species complex (FSSC) to undergo chromosome-level genome assembly using optical mapping. This assembly revealed the presence of 17 chromosomes, with 3 supernumerary chromosomes (LSCs) identified through pulsed-field gel electrophoresis analysis of various isolates (Coleman et al., 2009). Hence, the well-defined *F. vanettenii* 77-13-4 genome was often employed to compare with novel genome of *Fusarium* spp. (Williams et al., 2016; Hoh et al., 2022). These studies revealed core chromosomes (CCs) and lineage-specific chromosomes (LSCs) in *Fusarium* genomes. The LSCs are often associated with adaptation to diverse environments and implicated in virulence and pathogenicity (Coleman et al., 2009; Ma et al., 2013).

The pathways of pathogen dissemination and the infection progression of *Phalaenopsis* leaf yellowing remain poorly understood, making it challenging to pinpoint the optimal timing for disease control. To address this knowledge gap, it is imperative to develop and utilize specific primers for pathogen detection, elucidating its dissemination pathways. Whole-genome comparison serves not only to assess genetic similarities among related organisms but also to identify candidate genes of a species for primer design. For instance, Dobbs et al. (2020) developed specific primers for detecting highly virulent strains of *F. oxysporum* f. sp. *koae* by comparing genomes of strains with varying virulence levels. In addition, Short et al. (2017) conducted genome comparisons of seven species within the ambrosia *Fusarium* clade (AFC) to pinpoint specific sequences for primer design, enabling the discrimination and monitoring of AFC symbionts in ambrosia beetles across the United States.

In this investigation, *Fusarium phalaenopsidis* sp. nov. was established as the pathogen of *Phalaenopsis* leaf yellowing, supported by pathogenicity assay, phylogenetic analysis, and distinctive morphological characteristics. Through comparative genome analysis, the genomic architecture of this economically significant pathogen was unveiled. By comparing the proteomes of various *Fusarium* spp., unique genes for the development of specific primers targeting *F. phalaenopsidis* were identified. The specific primers were subsequently validated with multiple species within the FSSC. The results of the study provide fundamental information and a research tool for future investigations of the significant pathogen. The intended application of these specific primers is to facilitate pathogen surveillance and reveal pathogen dissemination pathways in orchid greenhouses.

## 2 Materials and methods

### 2.1 Isolation of fungal strains

Pathogens were isolated from leaf collars and basal stems. Several pieces of tissue (5 × 5 mm) cut from diseased or healthy plants were surface-sterilized in 1% or 3% sodium hypochlorite (NaClO) solution for 1 min, respectively, and then rinsed three times in sterile distilled water for 1 min. After drying, the pieces of tissue were placed on 2% water agar medium (WA) to reduce bacterial growth and incubated at room temperature for 1 week. Subsequently, all isolates were sub-cultured on potato dextrose agar (PDA, Becton Dickinson and Company Difco™) and further purified through single-spore isolation.

### 2.2 Morphology

The type strain FuZ10s was cultured on PDA and carnation leaf agar (CLA; 2% water agar sprinkled with 5 pieces of propylene oxide-fumigated carnation leaf) for morphological characterization (Leslie and Summerell, 2006). The micromorphology of the pathogen was examined and measured both on CLA and on artificially inoculated leaves of *Phalaenopsis* Sogo Yukidian “V3”. Colonies on PDA were cultured for 10 days at 25°C under 12/12 h light–dark cycles. Fungal materials were immersed in sterile distilled water and observed and measured using an upright microscope (Olympus BX53) equipped with differential interference contrast (DIC) for microscopic examination and a stereo microscope (Olympus SZX9) for macroscopic examination. The dimensions of each feature were recorded using a digital CMOS camera (E3ISPM Series) and calculated based on measurements from 30 randomly selected samples using Micrometrics SE Premium software (ACCU-SCOPE).

### 2.3 Genomic DNA extraction, amplification, and phylogenetic analysis

To harvest mycelia, each strain was cultured in 50 mL of yeast extract peptone dextrose broth (YEPD broth; 3 g yeast extract, 10 g peptone, 20 g dextrose/L) in an orbital shaker with 110 rpm at 25°C. After 3 days, the mycelia were collected with miracloth (Merck Millipore). The genomic DNA was extracted with the phenol-chloroform method (Green and Sambrook, 2012) and treated with RNase A (ProTECH, Taiwan) to remove RNA. Subsequently, the genomic DNA was precipitated with isopropanol, washed with 75% ethanol, dried in oven, and then dissolved in sterilized double distilled water.

The extracted DNA was subjected to PCR amplification. Four gene sequences of 19 studied isolates were amplified and used to construct phylogeny to infer organismal phylogeny, including translation elongation factor 1 alpha (*TEF1α*), internal transcribed spacer (ITS), large subunit of ribosomal ribonucleic acid (*LSU*), and the second largest subunit of the RNA polymerase II (*RPB2*). Gene sequences of the reference strains were retrieved from GenBank. To construct phylogeny, DNA sequences of each gene from all studied isolates and reference strains were aligned separately through MAFFT version 7 (Katoh et al., 2019) with default value parameter. Afterward, aligned gene sequences of each isolate were combined in the same order to generate a concatenated four-gene alignment dataset for phylogenetic analysis. Moreover, to enhance the phylogenetic resolution, sequences of three additional genes, calmodulin (*CAM*), ATP citrate lyase 1 (*ACL1*), and the largest subunit of the RNA polymerase II (*RPB1*), were incorporated with the four-gene alignment to generate a seven-gene alignment for phylogenetic analysis. Three isolates from the 19 isolates were selected for this further analysis. The primers used to amplify above genes are listed in Supplementary Table S1.

The phylogeny analyses were conducted through maximum likelihood (ML) (MEGA version 10) (Kumar et al., 2018) and Bayesian inference (BI) (Ronquist et al., 2012) method. The ML phylogeny was constructed with an appropriate model found by the Find Best DNA/Protein Models in MEGA version 10 and set 1,000 bootstraps for analysis. The BI phylogenies were constructed with MrBayes 3.2.7a and performed 1,000,000 times Markov chain Monte Carlo (MCMC). The trees were sampled every 1,000 generation (samplefreq = 1,000). The average standard deviation of slit frequencies of final analysis result was below 0.05, and the beginning 25% of sampled trees were discarded as burn-in.

### 2.4 Inoculation on *Phalaenopsis* leaf collar and detached leaves

Each isolate was cultured on PDA for 5 days at 25°C in 12/12 h light–dark cycles and prepared in spore suspension with a concentration of 10^5^ conidia/mL for inoculation. The plant materials used for inoculation were *Phalaenopsis* Sogo Yukidian “V3”. For inoculating detached leaves, the second and third mature leaves of 3.5-inch pots of “V3” plants were used and cleaned with 0.1% sodium hypochlorite solution. Next, the inoculation sites of leaves were wounded with the sterilized toothpick and dropped with 100 μL of conidium suspension. Subsequently, a 8 × 0.7 mm (diameter x thickness) filter paper disk (ADVANTEC) was covered on each inoculation site for moisture. Sterile water treatment was used as a negative control. The inoculated plants were placed in an airtight plastic box moistened with sterile water in dark at 25°C for 5 days. Leaf collar inoculation was performed on living *Phalaenopsis* “V3” plants in 2.5-inch pots with the same inoculation procedures described above. Three plants were used for a biological replicate, and three replicates were done.

### 2.5 Whole-genome sequencing, *de novo* assembly, and gene annotation

For whole-genome sequencing, the mycelia of FuZ10s were ground in liquid nitrogen to extract genomic DNA with the phenol-chloroform method (Green and Sambrook, 2012). After assessment of quality with 1.8–2.0 of O.D. 260/280 and ≥ 2.0 of O.D. 260/230, genomic DNA was used to generate sequencing library through NEBNext^®^ Ultra™ DNA Library Prep Kit for Illumina sequencing (NEB, United States). The Illumina NovaSeq 6,000 platform performed the 151 bp paired-end sequencing. For Nanopore sequencing (Oxford Nanopore Technology), the long DNA fragments were selected by BluePippin system (Sage Science, United States). The ends of DNA fragments were added A-tail through NEB Next Ultra II End Repair/dA-tailing Kit (NEB, United States). Subsequently, Qubit^®^ 3.0 Fluorometer (Invitrogen, United States) was employed to assess library fragment sizes, and Nanopore GridION X5 (Oxford Nanopore Technologies, United Kingdom) was applied to conduct sequencing.

The raw reads with mean qscore template <7 were removed, while high quality reads (Phred score > Q7) were selected by longQC v1.2.0 (Fukasawa et al., 2020) to assemble into the contigs performed by Flye v2.8.3 (Kolmogorov et al., 2019). Next, the contigs were polished by Racon v1.4.21 (Vaser et al., 2017) and to calibrate sequencing errors which were removed through Medaka v1.2.5^1^ and Homopolish v0.0.2 (Huang et al., 2021). For optimization of genome completeness, the contigs were polished with Illumina reads through Pilon v1.23 (Walker et al., 2014).

The sequence annotations of *Fusarium vanettenii* 77-13-4 from NCBI RefSeq and GenBank database were used as reference. Coding DNA sequences of genes were annotated against the reference by BRAKER2 (Brůna et al., 2021). Annotations of transfer RNA and ribosomal RNA were generated by tRNAscan-SE (Chan and Lowe, 2019) and Barrnap, respectively (Seemann, 2013). The functions of genes were searched using phmmer (Potter et al., 2018). The proteins not found in reference proteins were defined as hypothetical proteins. Gene ontology and Pfam domains of the coding DNA sequences were analyzed by InterProScan (Quevillon et al., 2005).

### 2.6 Comparative genomics of *Fusarium* spp.

To better understand the genomic information of *F. phalaenopsidis* FuZ10s, several genomic features were analyzed, including GC content, repeat region density, TE region density, gene density, and locations of secreted proteins, carbohydrate-active enzymes (CAZys), secreted CAZys, and effectors. *Fusarium phalaenopsidis* FuZ10s (Accession number: PRJNA785279) was compared with five *Fusarium* spp., namely, *Fusarium proliferatum* (Accession number: PRJNA576857), *Fusarium oxysporum* f. sp. *lycopersici* (Accession number: PRJNA948560), *Fusarium solani* FSSC 5 MPI-SDFR-AT-0091 (Accession number: PRJNA801211), *Fusarium vanettenii* 77-13-4 (Accession number: PRJNA51499), and *Fusarium ambrosium* NRRL 20438 (Accession number: PRJNA389173). To identify the repeat region and transposable element (TE) regions, the tools of repeatModeler2 (Flynn et al., 2020) and RepeatMasker (Chen, 2004) were used. The MUMmer v4.0.0 (Delcher et al., 2003) was used to identify the lineage-specific region (LS region) of *F. phalaenopsidis* that was further compared the LS regions of the five *Fusarium* species. All analyzed information was visualized with Circos (Krzywinski et al., 2009). Moreover, a chromosome-level comparison between *F. phalaenopsidis* FuZ10s and *F. vanettenii* 77-13-4 was performed by using MUMmer (Delcher et al., 2003). The genome regional linkage among these two species was visualized with Circos.

### 2.7 *In silico* prediction of secretome and effectors

Signal peptides of proteins were predicted with SignalP 5.0 (Almagro Armenteros et al., 2019), and transmembrane domains were predicted by TMHMM 2.0 (Krogh et al., 2001). Proteins possessing a signal peptide without transmembrane domains were considered as secreted proteins. The secreted proteins smaller than 350 amino acids were further predicted as effectors by EffectorP-fungi 3.0 (Sperschneider and Dodds, 2022). Pathogen–host interaction (PHI) database version 4.1 (Urban et al., 2022) was utilized to predict the putative PHI genes in *F. phalaenopsidis* FuZ10s genome. The prediction of secondary metabolites was analyzed via antiSMASH 6.0 (Blin et al., 2021).

### 2.8 Species-specific primer design and specificity assay

To obtain specific gene sequences for primer design, the total proteins of *F. phalaenopsidis* FuZ10s were compared with those of five others *Fusarium* spp. utilizing OrthoVenn2 (Xu et al., 2019). The five species included *Fusarium proliferatum* (Accession number: PRJNA576857), *Fusarium oxysporum* f. sp. *lycopersici* (Accession number: PRJNA948560), *Fusarium solani* FSSC 5 MPI-SDFR-AT-0091 (Accession number: PRJNA801211), *F. vanettenii* 77-13-4 (Accession number: PRJNA51499), and *F. ambrosium* NRRL 20438 (Accession number: PRJNA389173). Next, these gene sequences of either unique proteins of FuZ10s or proteins which did not be clustered (singletons) by OrthoVenn2 were screened using megablast in NCBI to select gene sequences without hits. These no-hit candidates were further divided based on the presence or absence of signal peptides using SignalP 5.0. The proteins with signal peptides were further predicted to be effector proteins or not using EffectorP 3.0, resulting in two protein groups: effector and non-effector proteins. In addition, the proteins without any signal peptides were further compared to virulence-related proteins in the pathogen–host interactions database (PHI-base) with E-value <10^4^. Afterward, these gene sequences of virulence-related proteins were screened for non-similarity using blastn in NCBI with E-value >10^−20^. Subsequently, proteins containing specific gene sequences were selected for specific primer design. Primer pairs were designed to amplify amplicons between 400 and 600 bp and were avoided with related sequences of organisms in *Fusarium oxysporum* species complex (FOSC). The specificity of primer pairs was evaluated by detecting 19 isolates of *F. phalaenopsidis*, 16 other members of FSSC (three isolates of *F. petroliphilum,* four isolates of *F. solani-melongenae,* five isolates of *F. falciforme*, two isolates of *F. solani sensu stricto*, and two isolates of *F. keratoplasticum*), six *Fusarium* species of non-FSSC (Supplementary Table S2), two orchid pathogens of *Phytophthora* (*Phytophthora nicotiana* and *Phytophthora palmivora*), and four species of saprophytic fungi (*Penicillium* sp., *Aspergillus* sp., *Trichoderma* sp., and *Rhizopus* sp.) commonly isolated from orchids.

For the PCR reactions, 12.5 μL of KAPA Taq ReadyMIX (KK1024, KAPA Biosystems), 9.5 μL of dH_2_O, 1 μL of the DNA sample (100 ng/μl), and 1 μL of each primer (10 μM) were mixed. The reaction conditions were as follows: initial denaturation at 95°C for 5 min, followed by 35 cycles of denaturation at 95°C for 30 s, and annealing and extension at 72°C for 30 s. The final extension step was performed at 72°C for 7 min. The annealing temperature was adjusted based on different primer pairs (Supplementary Table S1). The ITS sequences were amplified as DNA positive control. All primers used in this study are listed in Supplementary Table S1.

## 3 Results

### 3.1 Pathogen identification of *Fusarium* leaf yellows of *Phalaenopsis*

**Figure 1:**
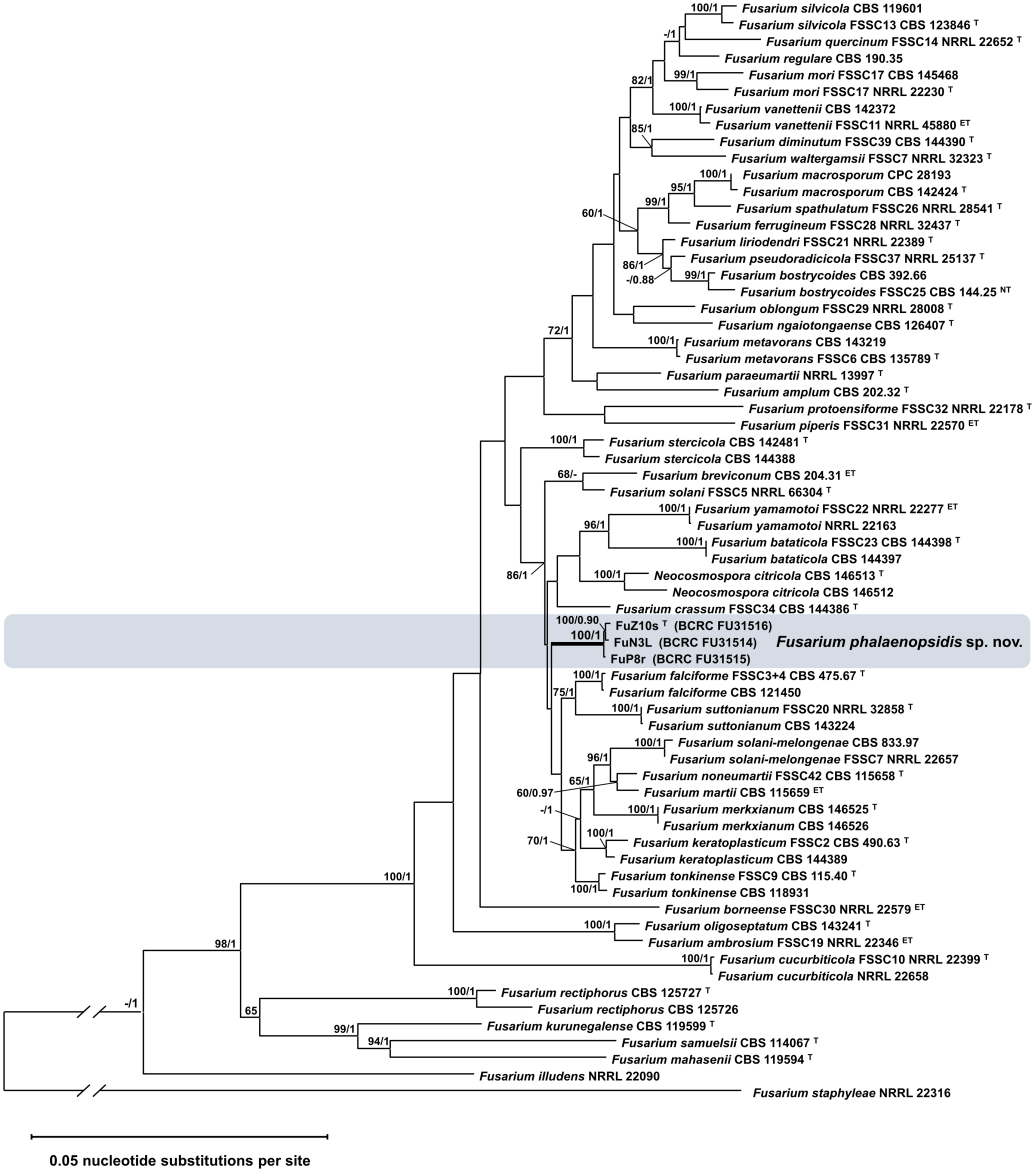
Phylogenetic analysis of *Fusarium phalaenopsidis* isolates. The phylogenetic tree inferred from concatenated sequences of ITS, TEF1, LSU, caM, acl1, RPB1, and RPB2 was generated by the maximum likelihood (ML) method with the general time reversible model. Values at nodes indicate bootstrap values >60% (1,000 replications) and the Bayesian posterior probability values >0.9. The tree is rooted to *Fusarium staphyleae* NRRL 22316. T, ex-type strain. ET, ex-epitype strain. The symbol “//” means abbreviation of 1 time of length based on the scale bar.

Sixteen *Fusarium* isolates obtained from symptomatic *Phalaenopsis* spp. displaying leaf yellows and three isolates collected from symptomless leaf blades in various locations across Taiwan were chosen for pathogenicity assays (Supplementary Table S2). Since the pathogen did not infect unwounded leaves, the inoculation was conducted on detached *Phalaenopsis* leaves. The results demonstrated that all 19 isolates induced necrotic symptoms. The leaf collar rot symptoms induced by isolate FuZ10s (= TNM F0036501) resembled those observed in the field affected by *Phalaenopsis* leaf yellowing (Supplementary Figure S1). To ascertain the taxonomic classification of these isolates within the FSSC, a phylogenetic analysis was performed using concatenated sequences from four genes: ITS (450 bp), *TEF1α* (458 bp), *LSU* (467 bp), and *RPB2* (1,289 bp). The resulting phylogenetic tree revealed that the 19 isolates formed a distinct, well-supported monophyletic clade with a bootstrap value of 100% and a Bayesian posterior probability of 1 (Supplementary Figure S2). Notably, this clade did not contain isolates from any recognized species and was closely related to the clade representing *F. solani sensu stricto* (FSSC 5). Moreover, to enhance the phylogenetic resolution, sequences from three additional genes, namely, *CAM* (361 bp), *ACL1* (367 bp), and *RPB1* (737 bp), were incorporated for three selected isolates. The resulting analysis indicated that these three isolates formed a cohesive monophyletic group with a bootstrap value of 100% and a Bayesian posterior probability of 1, distinct from any known species, suggesting a novel species within FSSC (Figure 1). Hence, the name *Fusarium phalaenopsidis* was proposed for this newly identified clade within FSSC, which encompasses isolate cpy01a, previously classified as *F. solani* f. sp. *phalaenopsis* (Chung et al., 2011).

### 3.2 Taxonomy of *Fusarium phalaenopsidis* sp. nov.

*Fusarium phalaenopsidis* C.L. Wang, & W.C. Tsao, sp. nov.
MycoBank: MB849930.
Etymology: Name refers to the former forma specialis.
Typification: Taiwan, Changhua County, from a necrotic leaf sheath with a yellowing leaf of *Phalaenopsis* sp., 14 July 2018, C. L. Wang, and W.C. Tsao. (holotype TNM F0036501 was deposited at the National Museum of Natural Science, Taiwan; ex-holotype strain BCRC FU31516 was preserved in the Bioresource Collection and Research Center, Taiwan).

#### 3.2.1 Description

##### 3.2.1.1 On artificially inoculated host leaves

**Figure 2:**
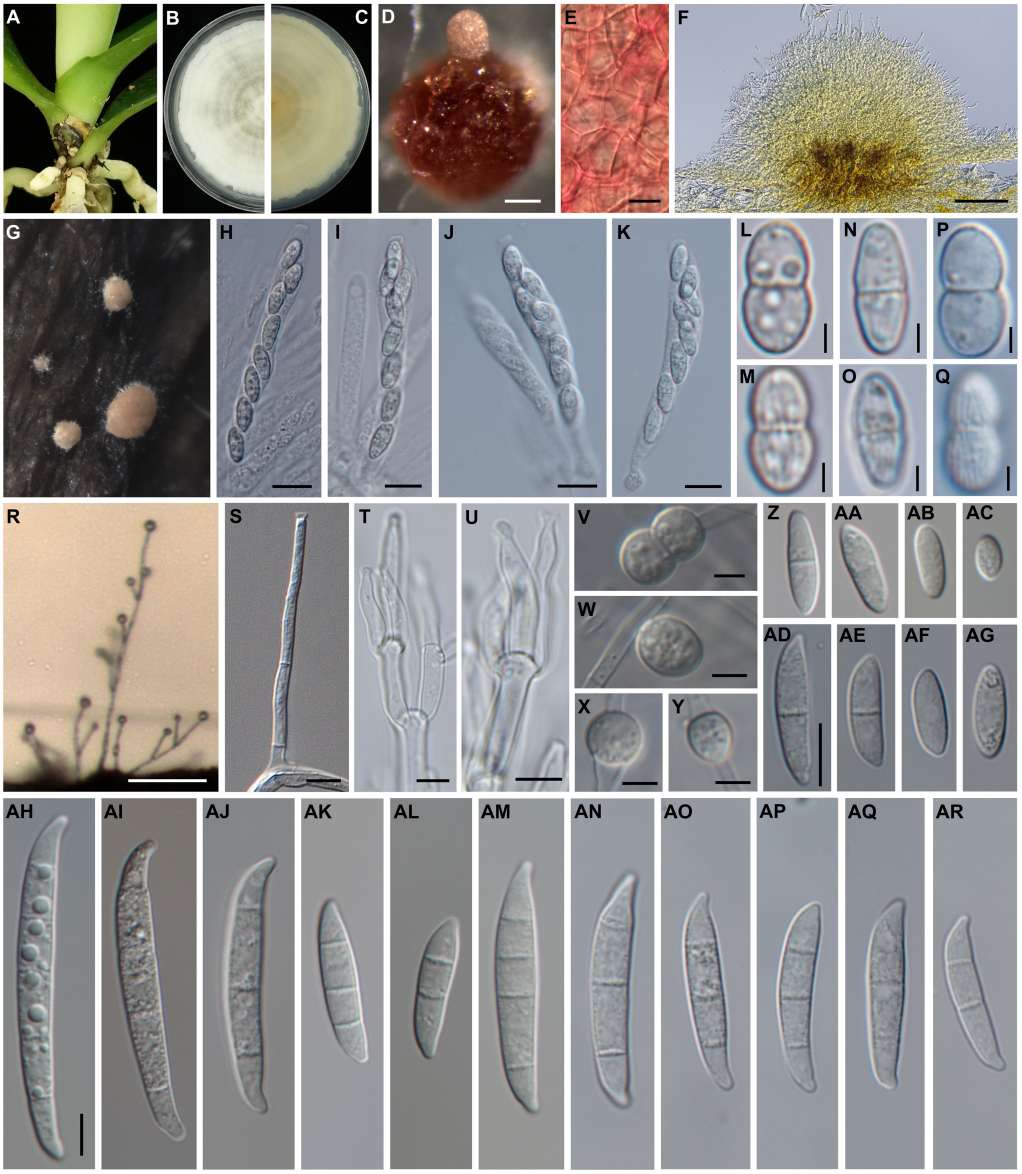
Morphology of *Fusarium phalaenopsidis* sp. nov. **(A)** The natural occurring symptoms on *Phalaenopsis* spp. caused by *F. phalaenopsidis.* **(B,C)** The upper and reverse sides of a colony on PDA. **(D)** A perithecium on CLA. **(E)** Perithecial wall cells. **(F)** A sectional view of a sporodochium. **(G)** Sporodochia on inoculated plants. **(H,I)** Asci from an inoculated plant. **(J,K)** Asci from CLA. **(L–O)** Ascospores from an inoculated plant. **(P,Q)** Ascospores from CLA. **(R)** Aerial conidiophores on an inoculated plant. **(S)** Aerial conidiophores on CLA. **(T,U)** Sporodochial conidiophores and phialides. **(V–Y)** Chlamydospores. **(Z–AC)** Microconidia from an inoculated plant. **(AD–AG)** Microconidia from CLA. **(AH–AL)** Sporodochial macroconidia from an inoculated plants. **(AM–AR)** Aerial macroconidia from CLA. Scale bar: 2.5 μm **(L–Q)**; 5 μm **(S–Y)**; 10 μm **(E,H–K,Z–AR)**; 100 μm **(D,F,R)**. **(Z–AG)** use same scale bar on **(AD)**; **(AH–AR)** use same scale bar on **(AH)**.

*Sexual and asexual morphs* produced on artificially inoculated leaves of *Phalaenopsis* Sogo Yukidian “V3”. *Perithecia* solitary to gregarious, superficial, rarely immersed in the host leaves, sometimes on stroma of sporodochia, orange to red or dark red, globose to obpyriform with a papillate ostiole; wall is rough and warty and composed of *textura globulosa* to *angularis. Asci* unitunicate, clavate with truncate apex, 71.8–104.2 × 9.0–11.6 μm (*n* = 13). *Ascospores* hyaline to pale brown, fusiform to ellipsoid, 10.4–12.7 × 4.6–6.2 μm (*n* = 22), 1-septate, distinctly constricted at the septum, often with slightly unequal size of two cells, longitudinally striated surface, obliquely uniseriate or biseriate at the apical one-third to half of asci. *Aerial conidiophores* mostly simple or branched with terminal monophialides; *Phialides* long cylindrical, 24.2–53.8 × 2.8–3.8 μm (*n* = 7), conidiogenous loci with flared collarette and inconspicuous periclinal thickening. *Aerial conidia* 0- to 1-septate; 0-septate conidia hyaline, ellipsoidal to obovoidal, 8.8–17.3 × 3.3–5.7 μm (*n* = 46); 1-septate conidia hyaline, ellipsoidal to slightly falcate, 12.5–22.9 × 4.0–6.1 μm (*n* = 34). *Sporodochia* white or cream to pale orange, abundant on host leaves, often dry, with stromata-like structure. *Sporodochial conidiophores* densely penicillate with compact matula, often arise from the stromatic-like cell, terminally bear single or whorl of two to three monophialides. *Sporodochial phialides* subulate to subcylindrical, 12.0–26.3 × 2.5–4.1 μm (*n* = 17), conidiogenous loci collarette flared and inconspicuous periclinal thickening. *Sporodochial conidia* 2- to 5-septate, mostly 4- to 5-septate; 2-septate conidia hyaline, clavate with slightly curved, 21.1–27.4 × 4.8–6.2 μm (*n* = 8); 3-septate conidia hyaline, clavate and falcate, 22.7–45.4 × 4.1–5.8 μm (*n* = 39); 4- to 5-septate conidia hyaline, falcate, almost straight in ventral side, slightly curved in dorsiventral side, apex slightly curved to hooked, basal cell papillate to well-developed, 4-septate: 34.9–52.0 × 4.2–6.2 μm (*n* = 29); 5-septate: 43.0–57.4 × 4.6–6.7 μm (*n* = 41). *Chlamydospores* not observed (Figure 2).

##### 3.2.1.2 On carnation leaf agar

*Sexual and asexual morphs* developed on carnation leaf agar. *Perithecia* solitary to gregarious, superficial, orange to red or dark red, globose to obpyriform with a papillate ostiole, wall is rough and warty and comprise *textura globulosa* to *angularis*. *Asci* unitunicate, clavate, apex flat, 56.7–98.1 × 8.1–11.6 μm (*n* = 30)*. Ascospores* are hyaline to pale brown, fusiform to ellipsoid, 9.9–13.3 × 4.5–6.7 μm (*n* = 36), 1-septate, obviously constricted at the septum with slightly unequal sizes of two cells, longitudinally striated surface, obliquely uniseriate or biseriate at apical one-third to half of asci. *Aerial conidiophores* mostly simple or branched with monophialides*; phialides* mostly cylindrical or slightly subulate, flared collarette and inconspicuous periclinal thickening, 38.6–79.6 × 2.6–4.2 μm (*n* = 35). *Aerial microconidia* 0- to 1- septate, 0-septate hyaline, often ellipsoidal to obovoidal, 6.0–17.7 × 2.5–4.7 μm (*n* = 53); 1-septate conidia hyaline, ellipsoidal straight curved, 13.8–24.4 × 3.6–5.4 μm (*n* = 39); *macroconidia* 2- to 5-septate, mostly 3-septate, hyaline, almost straight and slightly curved at the ends, apex blunt to slightly beaked, basal cell papillate; 2-septate *macroconidia*: 20.5–31.3 × 4.4–6.2 μm (*n* = 10), 3-septate *macroconidia*: 23.8–47.7 × 4.3–6.6 μm (*n* = 37), 4-septate *macroconidia*: 34.7–49.0 × 5.2–7.2 μm (*n* = 17), 5-septate *macroconidia*: 46.9–52.7 × 6.1–6.9 μm (*n* = 3). *Sporodochia* are not observed on CLA. *Chlamydospores* are hyaline or pale brown, thick-walled, globose to subglobose, single or paired, terminal or intercalary, abundantly produced, 6.3–9.8 × 5.0–9.2 μm (*n* = 11) (Figure 2).

*Colonies* achieve diameter 61.8 to 63.1 mm at 7 day on PDA with an average growth rate of 4.4 to 4.5 mm/day at 25°C, 12 h light photoperiods, white to cream, upper side margin entire with circular concentric rings or not, cream to buff in underside.

##### 3.2.1.3 Notes

Although *F. phalaenopsidis* was placed in a clade of 86% bootstrap value and 1 Bayesian posterior probability that encompasses 14 known species in the FSSC phylogenetic tree (Figure 1), there were some morphological characters distinguishing *F. phalaenopsidis* from others in this clade. For instance, most of species in this clade did not record sexual stage or maybe heterothallic, except for *F. yamamotoi*, *F. solani-melongenae*, *F. keratoplasticum* (Sandoval-Denis and Crous, 2018; Sandoval-Denis et al., 2019; Crous et al., 2021; Guarnaccia et al., 2021), and *F. phalaenopsidis* recorded in this study. Among the four species, the sporodochial conidia of *F. yamamotoi* (6-septate of largest, overall: 34.5–78.5 × 4–6.5 μm) and *F. solani-melongenae* (9-septate of largest, overall: 28.0–95.5 × 4.5–7.5 μm) revealed longer and more septa than *F. phalaenopsidis* (5-septate of largest, overall: 21.1–57.4 × 4.8–6.7 μm). Although *F. keratoplasticum* (overall: 13.2–60.1 × 2.8–8.2 μm) was closed to *F. phalaenopsidis* in sporodochial conidia, its ecologic niches of animal substrates (Short et al., 2013) were distinct from *F. phalaenopsidis.* In addition, the two species were phylogenetically distant from each other in this clade. The *TEF1α* gene sequence of *F. phalaenopsidis* ex-type strain (BCRC FU31516) was only 93.7% identical to that of *F. keratoplasticum* (CBS 490.63) with 10 bp polymorphisms, 13 bp insertions, and 4 bp deletions. Notably, *F. phalaenopsidis* did not generate sporodochia on CLA. Stroma-like structures in the described sporodochia developing on the inoculated *Phalaenopsis* leaves were a distinct feature as well.

### 3.3 Genome structure of *Fusarium phalaenopsidis* FuZ10s

**Figure 3:**
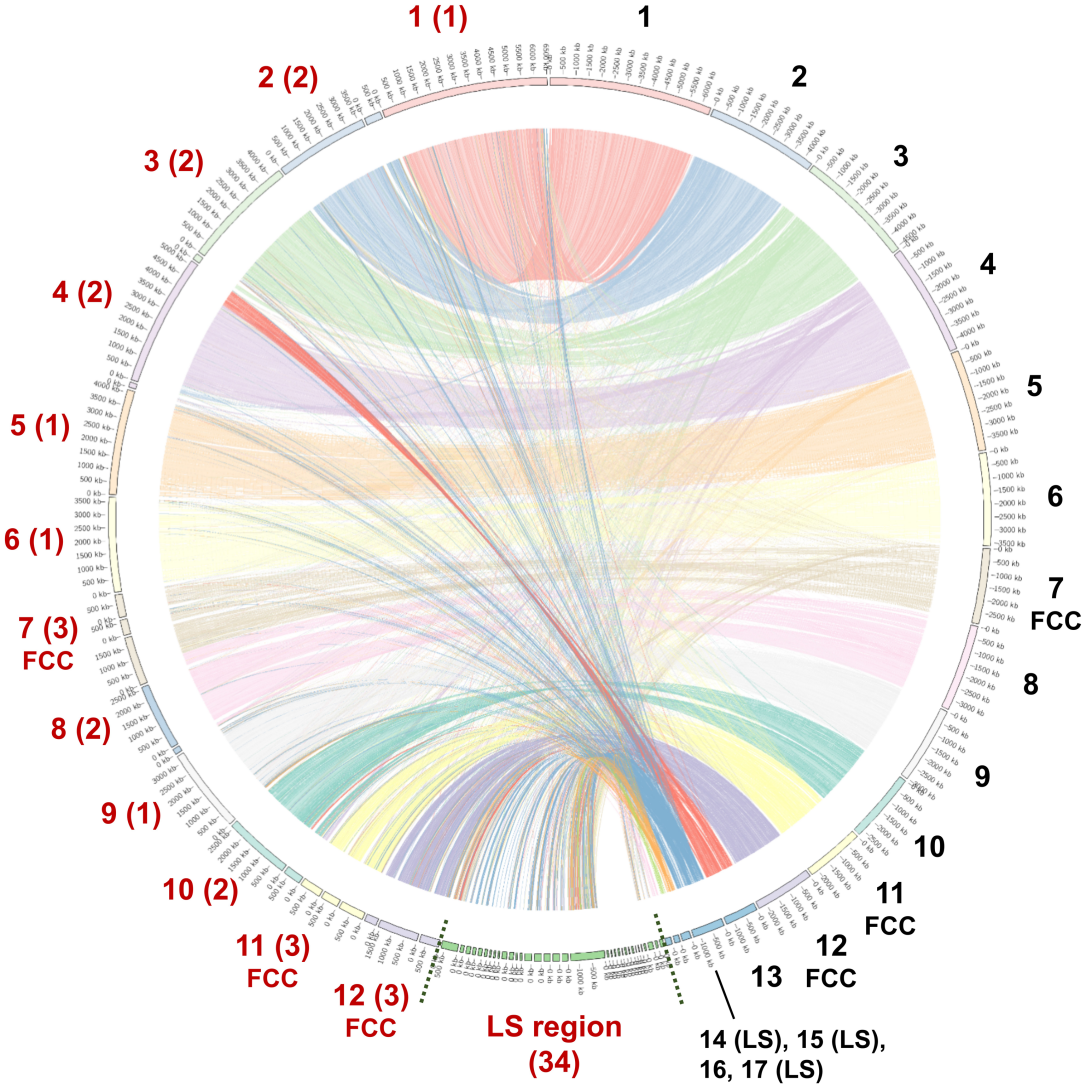
Genome regional linkage map between *Fusarium phalaenopsidis* FuZ10s (Fp) and *Fusarium vanettenii* 77-13-4 (Fv). The map is a comparing synteny between Fp and Fv genomes. The red numbers in addition to the map indicated inferred Fp chromosomes and lineage-specific (LS) region, whereas the black numbers indicate Fv chromosomes. Fast core chromosomes (FCCs) and LS region of two genomes are indicated. The numbers in parentheses indicate the contig number comprising each chromosome of *F. phalaenopsidis*.

To facilitate future molecular investigations of *F. phalaenopsidis*, the holotype strain FuZ10s was subjected to whole-genome sequencing. The *de novo* assembly of the FuZ10s strain genome combined the sequence databases from Nanopore (long-read data) and Illumina (short-read data) sequencing and produced a total length of 52,271,178 bp (52.2 Mb) distributed across 57 contigs, with an N50 length of 3,659,495 bp (3.65 Mb) (Table 1). The BUSCO assessment yielded a score of 98.80%, indicating high quality and completeness of the sequenced FuZ10s genome. In the synteny analysis, the genome of *F. phalaenopsidis* FuZ10s was compared with the genome of *F. vanettenii* 77-13-4, which has been well assembled at the chromosome level. This comparison revealed 12 mapped regions that exhibited significant synteny with the 12 CCs of *F. vanettenii* 77-13-4 (Figure 3). Consequently, these 12 mapped regions were designated as CCs, while the contigs that did not align with the CCs of *F. vanettenii* 77-13-4 were assigned as a lineage-specific (LS) region of *F. phalaenopsidis*.

**Table 1:** Genome *de novo* assembly and gene functional annotation statistics for *Fusarium phalaenopsidis* FuZ10s generated with Nanopore sequencing polished with Illumina reads.

Assembly	
Sum (bp)	52,271,178
GC (%)	50.89
Contig number (> = 500 bp)	57
Largest contig (bp)	6,521,254
Mean contig (bp)	917,038
Minimal contig (bp)	8,675
N50 (bp)	3,659,495
N75 (bp)	1,590,632
Coverage (fold)	111
BUSCO (%)	98.80%

Functional annotation	
Total predicted genes	14,633
rRNA number	84
tRNA number	305
KEGG hit	5,249
GO hit	7,165
Predicted secreted proteins^a^	1,162
Effector candidates^b^	267

a Secreted proteins predicted by SignalP 5.0 and TMHMM 2.0, respectively, contain signal peptides and do not contain transmembrane domains.

b Secreted proteins less than 350 amino acids were predicted as effectors by EffectorP-fungi 3.0.

### 3.4 Functional annotation of *Fusarium phalaenopsidis* FuZ10s genome

**Figure 4:**
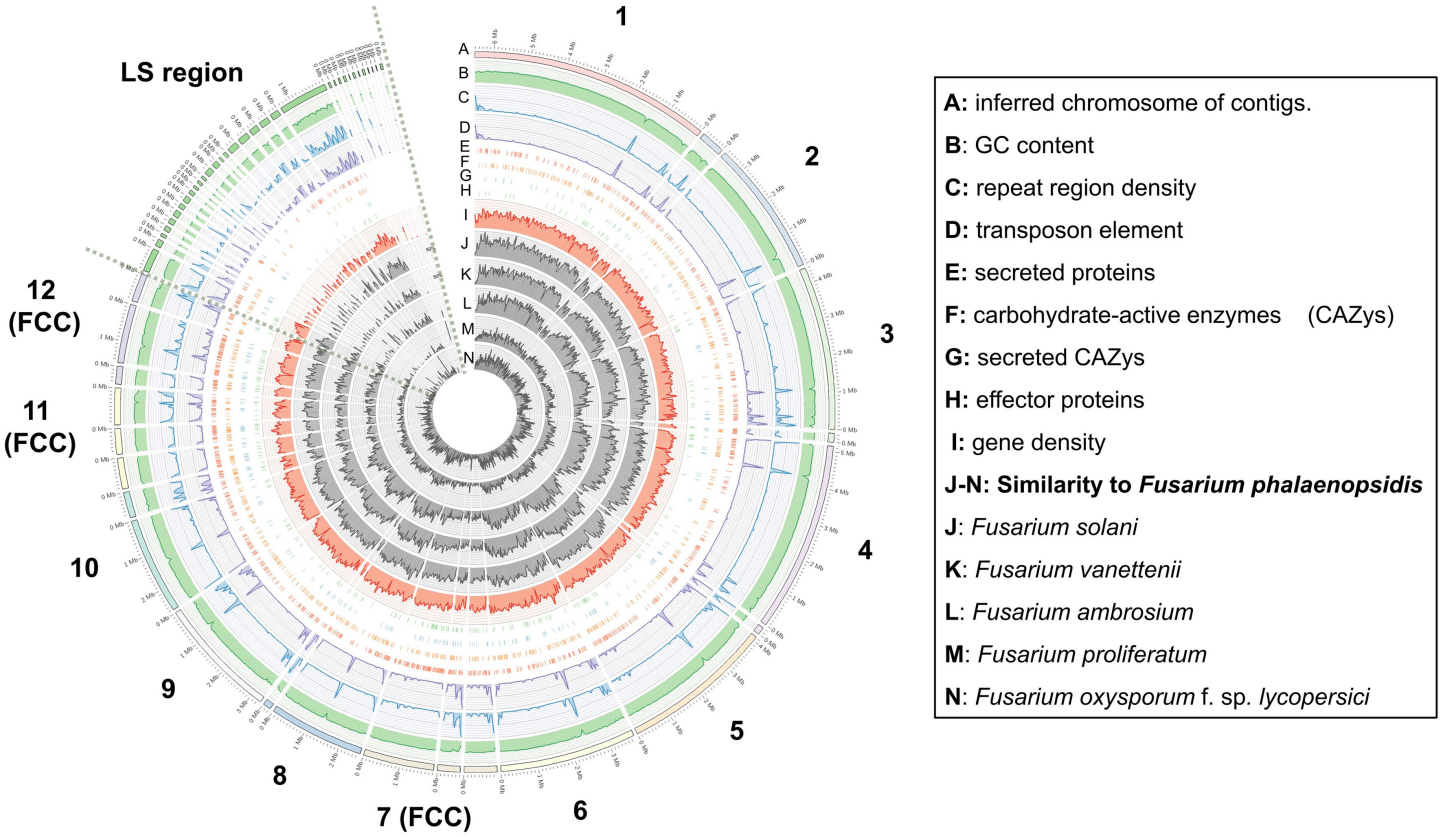
Distribution and abundance of featured elements on each chromosome and lineage-specific region of *Fusarium phalaenopsidis*. FCC, fast core chromosome; LS region, lineage-specific region.

**Figure 5:**
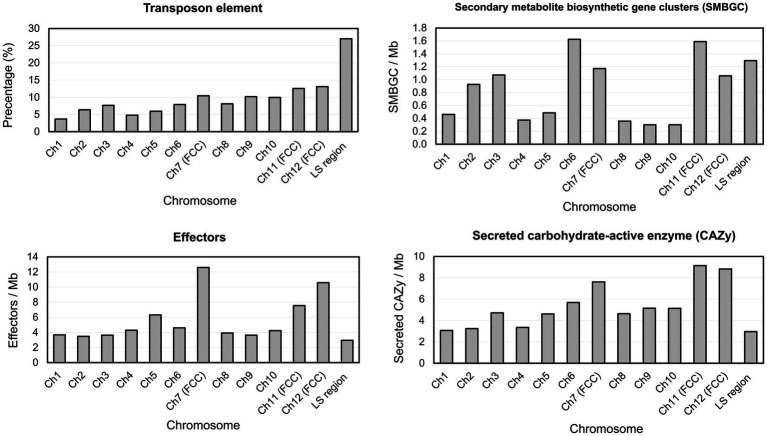
Percentage of transposon element and density of virulence-related genes on each *Fusarium phalaenopsidis* chromosome. FCC, fast core chromosome; LS region, lineage-specific region.

Gene prediction identified a total of 14,633 genes in the assembled genome of strain FuZ10s. To delve deeper into genes associated with pathogenesis, analysis was conducted on the 12 CCs and the LS region of *F. phalaenopsidis* FuZ10s, focusing on secreted proteins, transposon elements, and secondary metabolite biosynthetic gene clusters (Figures 4, 5; Supplementary data sheets S1–S4). Among these, 1,162 genes were predicted to encode secreted proteins based on the presence of signal peptides and the absence of transmembrane domains (Figure 4; Supplementary data sheets S2–S4). In addition, analysis of secreted proteins smaller than 350 amino acids revealed 267 effector candidates of *F. phalaenopsidis* (Supplementary data sheets S2–S4). Notably, the LS region exhibited a significantly higher abundance of transposon elements compared to other CCs (Figure 5). Chromosomes 6 and 11, along with the LS region, displayed an enrichment of predicted secondary metabolite biosynthetic gene clusters relative to other chromosomes, with the LS region containing all types of predicted secondary metabolite biosynthetic gene clusters. In addition, 12 out of 43 predicted secondary metabolite biosynthetic gene clusters in the genome were predicted to generate known compounds, while the remaining ones were predicted to produce unknown compounds (Supplementary Table S3). Gene ontology analysis of the LS region indicated enrichment for transmembrane transporter activity, DNA and ATP/ADP binding activity, as well as oxidoreductase, monooxygenase, and hydrolase activity among 307 out of 900 total proteins (Supplementary data sheet S5). Moreover, the LS region contained only 16 effector genes (Supplementary data sheet S4), of which 4 effectors with predicted functional domains in Pfam (Sonnhammer et al., 1997) were a PAN domain-containing protein (g6841), a hydrophobin (g13479), a glycosyl hydrolase family 61 (g6084), and a peptidase A4 family (g6210). Furthermore, virulence-related genes, including effectors and secreted carbohydrate-active enzymes (CAZys), were found to be enriched in chromosomes 7, 11, and 12 (Figure 5), being consistent with the characteristics of the “fast core chromosomes” observed in FSSC (Hoh et al., 2022). These secreted proteins encompassed glycosyl hydrolases (GH), necrosis-inducing proteins (NPP), cerato-platanin, and tuberculosis necrotizing toxin (TNT)-like proteins, which could potentially contribute to the necrotic symptoms associated with *Phalaenopsis* leaf yellowing (Table 2).

**Table 2:** Necrosis-related proteins in fast core chromosome (FCC).

Chromosome	Contig ID	Gene ID	Gene Name	Pfam
Ch7	contig_32328	g2201	Hypothetical protein	Necrosis-inducing protein (NPP1)
	contig_32288	g4622	Hypothetical protein	Necrosis-inducing protein (NPP1)
	contig_32328	g2190	Hypothetical protein	Glycosyl hydrolases family 43
	contig_32328	g2238	Glycoside hydrolase family 31	N-terminal barrel of NtMGAM and CtMGAM, maltase-glucoamylase; Galactose mutarotase-like; Glycosyl hydrolases family 31
	contig_32288	g4415	Hypothetical protein	Glycosyl hydrolases family 43
	contig_32288	g4425	GH16 domain-containing protein	Glycosyl hydrolases family 16
	contig_32288	g4630	Hypothetical protein	Glycosyl hydrolases family 28
	contig_32288	g4794	Hypothetical protein	Glycosyl hydrolases family 43
	contig_32372	g12763	Hypothetical protein	Cerato-platanin protein
	contig_32372	g12729	Glycoside hydrolase family 17	Glycosyl hydrolases family 17
	contig_32372	g12823	Polygalacturonase	Glycosyl hydrolases family 28
	contig_32372	g12841	Endo-1,4-beta-xylanase	Glycosyl hydrolases family 11
Ch11	contig_32282	g6422	Hypothetical protein	Glycosyl hydrolases family 43
	contig_32282	g6482	Hypothetical protein	Glycosyl hydrolases family 28
	contig_32282	g6675	Hypothetical protein	Glycosyl hydrolases family 28
	contig_32274	g10964	Chitinase	Chitin recognition protein; GH family 18; LysM domain
	contig_32276	g11108	Hypothetical protein	Glycosyl hydrolases family 43
	contig_32276	g11142	GH16 domain-containing protein	Glycosyl hydrolases family 16
Ch12	contig_32308	g7031	Hypothetical protein	Glycosyl hydrolases family 43
	contig_32308	g7066	Hypothetical protein	Glycosyl hydrolases family 28
	contig_32306	g1642	Hypothetical protein	Glycosyl hydrolases family 28
	contig_32306	g1912	Hypothetical protein	GH family 2, sugar binding, TIM barrel domain
	contig_32306	g1928	Beta-galactosidase	Beta-galactosidase, jelly roll domain; GH family 35
	contig_32306	g1937	Arabinan endo-1,5-alpha-L-arabinosidase	Glycosyl hydrolases family 43
	contig_32376	g11431	Hypothetical protein	Glycosyl hydrolases family 43
	contig_32376	g11449	Hypothetical protein	Glycosyl hydrolases family 28
	contig_32306	g1657	Hypothetical protein	Necrosis-inducing protein (NPP1)
	contig_32306	g1734	Hypothetical protein	Necrosis-inducing protein (NPP1)
	contig_32306	g1743	Hypothetical protein	Tuberculosis necrotizing toxin

### 3.5 Development of *Fusarium phalaenopsidis*-specific primers

**Figure 6:**
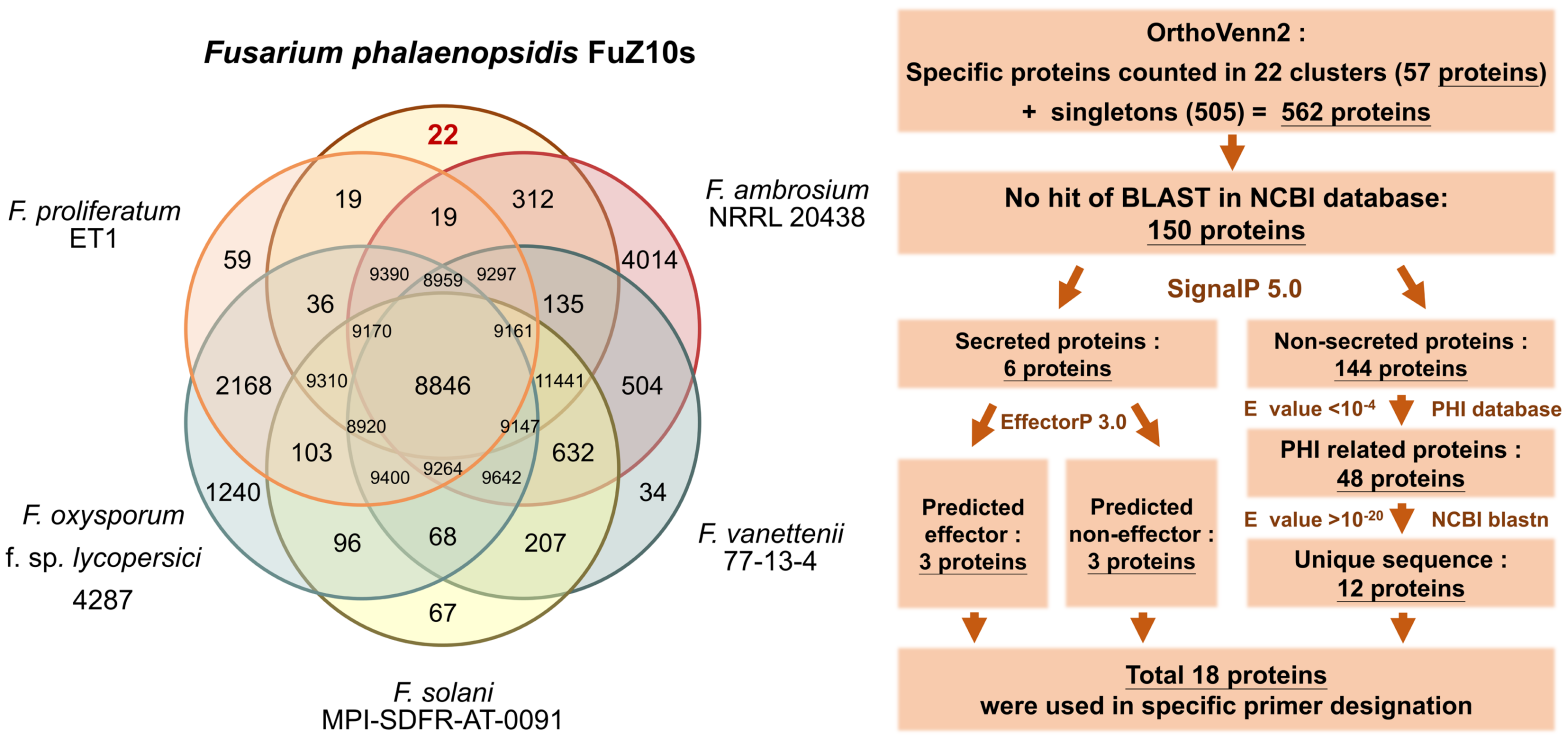
Comparison of orthologues protein clusters and the flowchart of process to screen proteins for specific primer designation.

To identify candidate proteins for developing specific primers of *F. phalaenopsidis*, OrthoVenn2 was employed to compare the total proteins of *F. phalaenopsidis* FuZ10s with those of five *Fusarium* species, including three species of FSSC (*F. ambrosium* NRRL 20438, *F. vanettenii* 77-13-4, and *F. solani* MPI-SDFR-AT-0091), one species of *F. oxysporum* species complex (*F. oxysporum* f. sp. *lycopersici* 4,287), and one species of *F. fujikuroi* species complex (*F. proliferatum* ET1) (Figure 6). This analysis identified 57 proteins in 22 clusters that were unique to FuZ10s among the six species. In addition, 505 singleton proteins were not clustered in OrthoVenn2. Subsequently, a total of the 562 gene sequences of proteins were screened by comparing them with the GenBank database through NCBI megablast (with the setting of “Highly similar sequences” at NCBI), revealing that 150 gene sequences had no matches (no hits) for any similar genes.

These 150 proteins were categorized based on the presence or absence of signal peptides. Among them, six proteins, including three predicted effectors and three non-effectors, were predicted to be secreted, while the remaining 144 proteins were not. The 144 proteins were then compared with the PHI-base, revealing 48 virulence-related proteins with E-value <10^−4^. Further comparison of the gene sequences of these 48 proteins with the GenBank database through the BLASTN program (with the setting of “Somewhat similar sequences” at NCBI) unveiled 12 unique genes of virulence-related proteins with E-value >10^−20^. Finally, a total of 18 gene sequences of proteins, including the 12 virulence-related proteins and the six secreted proteins, were selected for designing specific primers (Table 3; Supplementary Table S1 and screening process in Supplementary Table S4).

**Table 3:** Genes of *Fusarium phalaenopsidis* used in primer design and primer specificity.

Gene ID	Annotation	GO –molecular function	Amplicon size^b^ (bp)	Annealing temperature^c^ (°C)	Primer specificity^d^
Effector^a^					
g14599	Hypothetical protein	–	309	62	Na
g1689	Carboxypeptidase	Serine-type carboxypeptidase activity	662	60	Y
g6406	Hypothetical protein	–	402	58	Na
Non-effector					
g12851	ANK_REP_REGION domain-containing protein	Protein binding	418	58	Na
g12868	ANK_REP_REGION domain-containing protein	Protein binding	638	58	Na
g6699	ANK_REP_REGION domain-containing protein	–	707	56	Na
Non-secreted PHI-associated protein					
g10855	Serine/threonine protein kinase-like protein	–	712	56	Na
g11269	Ankyrin-2-like	–	628	56	Nb
g11441	Similar to transcription factor steA	DNA binding; zinc ion binding	487	56	Nb
g13046	SRP54 domain-containing protein	GTP binding	552	60	Nb
g13762	Probable cutinase transcription factor 1 beta	DNA binding; zinc ion binding	832	56	Nb
g13852	Fungal specific transcription factor	–	499	56	Na
g6259	Hypothetical protein	–	474	56	Nb
g6260	Fungal_trans domain-containing protein	DNA binding; zinc ion binding	436	56	Nb
g6345	ANK_REP_REGION domain-containing protein	–	557	58	Y
g6915	Related to *P. aeruginosa* anthranilate synthase component II	–	558	58	Na
g8218	Nitrogen assimilation transcription factor nira	–	411	58	Na
g9170	Hypothetical protein	–	318	58	Na

a Effector proteins were determined by EffectorP 3.0.

b The size of amplicons from designed primers.

c The annealing temperature of designed primers utilized in PCR program.

d The symbol “Na” indicates that the primers amplified the specific band from partial isolates of *Fusarium phalaenopsidis*; “Nb” indicates that the primers amplified the specific band from *F. phalaenopsidis* and non-target fungi; “Y” indicates that the primers amplified the specific band from all isolates of *F. phalaenopsidis* but not from non-target fungi.

**Figure 7:**
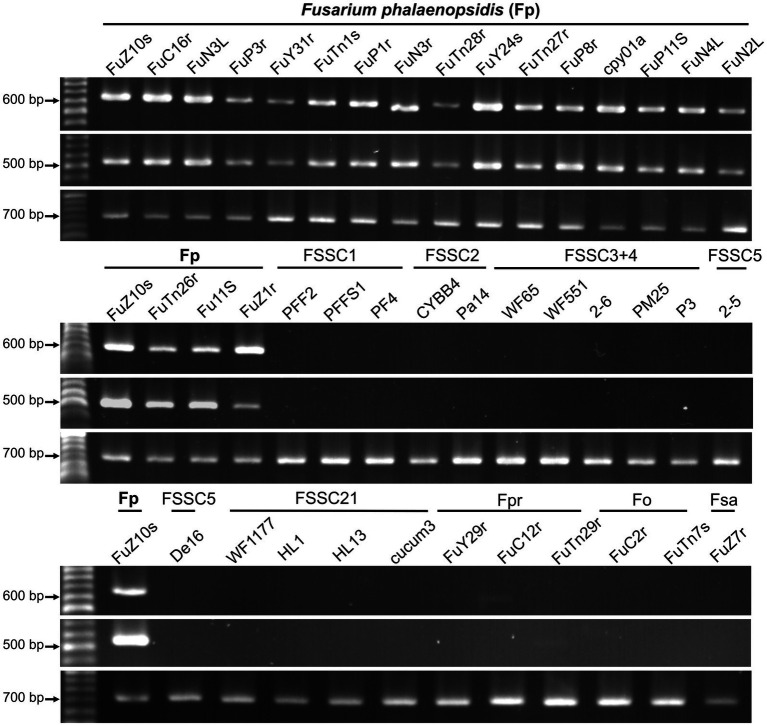
Primer applicability and specificity assays. The primer sets used in assays are FphSP1F/FphSP1R (upper panel) and FphSP6F/FphSP6R (middle panel) for detecting *Fusarium phalaenopsidis* (Fp) and V9G/ITS4 (lower panel) for verifying quality of DNA from all tested isolates. FSSC1, *Fusarium petroliphilum*; FSSC2, *Fusarium keratoplasticum*; FSSC3 + 4, *Fusarium falciforme*; FSSC5, *Fusarium solani*; FSSC21, *Fusarium solani-melongenae*; Fpr, *Fusarium proliferatum*; Fo, *Fusarium oxysporum*; Fsa, *Fusarium sacchari.*

Eighteen primer pairs were designed based on the gene sequences of these proteins, with 35 primers targeting the coding region of these gene sequences, while the forward primer of gene g1689 was designed in the non-coding region. An applicability assessment indicated that primer pairs of 10 genes only amplified target bands from partial tested *F. phalaenopsidis* isolates (Table 3). The remaining eight primer pairs that passed the applicability assessment were further used to assess specificity with a total of 28 non-target species of fungi or fungus-like organisms, including 16 other members of FSSC, six *Fusarium* species of non-FSSC (Supplementary Table S2; Supplementary Figure S3), two *Phytophthora* spp., and four species of fungi commonly isolated from *Phalaenopsis* spp. The results indicated that only two primer pairs designed from genes g1689 and g6345 could amplify specific bands from all *F. phalaenopsidis* but not from non-target fungi (Table 3; Figure 7; Supplementary Figure S4). These primers were named FphSP1F and FphSP1R for gene g1689 and FphSP6F and FphSP6R for gene g6345. To further assess sensitivity, the two pairs of specific primers were used to amplify *F. phalaenopsidis* FuZ10s DNA at different concentrations. The results suggested that these primer pairs could amplify specific bands at least at a DNA concentration of 100 pg./μL (Supplementary Figure S5).

## 4 Discussion

*Phalaenopsis* leaf yellowing was first reported in Taiwan in 2010 and attributed to *F. solani* (Su et al., 2010). Later, the pathogen was further recognized as *F. solani* f. sp. *phalaenopsis* based on its host specificity to *Phalaenopsis* (Chung et al., 2011). In 2016, the presence of *F. solani* f. sp. *phalaenopsis* in Australia was verified and associated with orchid species of *Cymbidum*, *Cattleya,* and *Laelia* by sequencing the cultures collected in 1996, suggesting a wider host range of the pathogen (Laurence et al., 2016). On the other hand, *F. solani* isolates identified prior to the study of Schroers et al. (2016) or without comparison with FSSC 5 should be considered as members of *F. solani sensus lato*, a species complex encompassing isolates in polyphyletic clades. Thus, although *F. solani* has been reviewed to cause various symptoms on orchids, including *Phalaenopsis*, *Cymbidium*, *Dendrobium*, and *Miltonia*, in several countries, such as Korea, Japan, Malaysia, and USA (Hawaii) (Srivastava et al., 2018), it is still obscure if those pathogens are *F. solani* f. sp. *phalaenopsis*. Moreover, there May be contradictions in the use of “forma specialis,” leading to misunderstandings about the host pathogenicity (Šišić et al., 2018a). For instance, *Fusarium solani* f. sp. *pisi* (current name *F. vanettenii*) is named based on its pathogenicity on *Pisum sativum*. However, the virulence test on 62 species of legumes indicated that *F. vanettenii* is capable of infecting 33 species of legumes, implicating that forma specialis system cannot well define the pathogen (Šišić et al., 2018b). Our phylogenetic analysis indicates that all *Phalaenopsis* leaf yellowing pathogens, including *F. solani f.* sp. *phalaenopsis* cpy01a (Chung et al., 2011) (Supplementary Figure S2), were clustered in a monophyletic clade with strongly supported and did not include any known species, indicating that phalaenopsis leaf yellow pathogen is a novel phylogenetic species. To stabilize the nomenclature status of the economic important pathogen, *F. phalaenopsidis* was introduced as a new species based on the molecular information and morphological features.

Hoh et al. (2022) sequenced six genomes of five FSSC species in chromosome level to illustrate chromosome structures of species in FSSC. Among them, *Fusarium* sp. Ph1 isolate obtained from tissues of *Phalaenopsis* leaf yellowing is likely a member of *F. phalaenopsidis*. The research also explored transcriptomes of *F. falciforme* and *F. keratoplasticum* infecting sea turtles, without delving into the *F. phalaenopsidis* aspect. In the current study, the genome of *F. phalaenopsidis* holotype strain FuZ10s (BCRC FU31516) was sequenced and further compared with *F. vanettenii* 77-13-4. The results suggested that *F. phalaenopsidis* possess 12 core chromosomes and the LS region. Three FCCs 7, 11, and 12 of FSSC were delineated which harbor higher FSSC-specific orthologues when compared with non-FSSC *Fusarium* species including *F. oxysporum*, *F. graminearum*, and *F. fujikuroi* (Hoh et al., 2022). These FCCs exhibit an enrichment in the number of effectors, CAZys, and SMBGCs (Hoh et al., 2022), corresponding to features observed in *F. phalaenopsidis* FuZ10s FCCs (Figure 5). Furthermore, some effectors within *F. phalaenopsidis* FCCs are potential in contributing to the necrotic symptoms of *Phalaenopsis* leaf yellowing, such as necrosis-inducing proteins (NPP) (g2201, g4622, g1657, and g1734) (Gijzen and Nürnberger, 2006), cerato-platanin proteins (g12763) (Pazzagli et al., 2006), and tuberculosis necrotizing toxin (TNT)-like proteins (g1743) (Sun et al., 2015) (Table 2). Many glyscosyl hydrolases (GHs) in FCCs belong to enzyme families of GH11 (g12841), GH17 (g12729), and GH28 (g4630, g12823, g6482, g6675, g7066, g1642, and g11449) which are likely associated with plant cell wall degradation and virulence (Brito et al., 2006; Have et al., 1998; Liu et al., 2023; Kars et al., 2005). For instance, *Magnaporthe oryzae* Ebg1, an exo-*β*-1,3-glucanase belonging to the GH17 family, is crucial for pathogenicity through suppressing plant immunity during the invasive hyphal growth stage within rice cells (Liu et al., 2023). In addition, *Botrytis cinerea* endopolygalacturonase BcPG1 and BcPG2 belonging to GH28 are required for virulence on tomato (Have et al., 1998; Kars et al., 2005). *Botrytis cinerea* xylanase BcXyl1 belonging to GH11 is a virulence factor on tomato leaves and grape fruit (Brito et al., 2006). In addition, gene g12763 located on FCC 7 was predicted to encode a cerato-platanin protein that has been implicated as an elicitor, inducing cell death in hosts (Pazzagli et al., 2006) and contributing to virulence in *Sclerotinia sclerotiorum* (SsCP1), *B. cinerea* (BcSpl1), and *F. oxysporum* f. sp. *cubense* Tropical Race 4 (Frías et al., 2011; Yang et al., 2018; Liu et al., 2019). Moreover, four genes resided on FCC 7 (g2201 and g4622) and FCC 12 (g1657 and g1734) were predicted to encode the NPP1 domain and were considered members of a large protein family capable of inducing cell death in host. They also serve as virulence factors, accelerating disease progression (Gijzen and Nürnberger, 2006). Interestingly, the gene g1743 located on FCC 12 was predicted to encode a tuberculosis necrotizing toxin (TNT) domain-containing protein which acts as necrosis factor of human bacteria pathogen *Mycobacterium tuberculosis* (Sun et al., 2015). However, the function of TNT in plant fungal pathogens remains largely unknown. Further investigation into the roles of these potential NPPs, GHs, cerato-platanin proteins, and cerato-platanin proteins in the pathogenicity of *F. phalaenopsidis* is warranted.

In addition to high percentage of the transposon elements, the LS region of *F. phalaenopsidis* encompasses protein enrichment of monooxygenase activity (25 proteins), oxidoreductase activity (49 proteins), and transmembrane transporter activity (58 proteins) (Supplementary data sheet S5). Research studies revealed that monooxygenases, dehydrogenases, and transmembrane transporters serve as virulence factors in plant fungal pathogen (Aliyu et al., 2019; Coleman, 2016; Enkerli et al., 1998; Klose and Kronstad, 2006; Liu et al., 2017; Roohparvar et al., 2007). For instance, the maackiain detoxification gene 1 (MAK1), which encodes a flavin adenine dinucleotide (FAD)-containing monooxygenase, is located on the LS chromosome 14 of *F. vanettenii*. The enzyme metabolizes maackiain to a less toxic form, suggesting that detoxification via this monooxygenase is a partial determinant of the pathogen’s ability to infect chickpea (Coleman, 2016; Enkerli et al., 1998). Dehydrogenases constitute a group of oxidoreductase. The short-chain acyl-CoA dehydrogenases *MoSCAD1* and *MoSCAD2* of *Magnaporthe grisea* were shown to play roles in stress tolerance, growth, conidiation, and pathogenicity (Aliyu et al., 2019). *Ustilago Maydis MFE2* encoding a dehydrogenase protein contributed virulence in corn (Klose and Kronstad, 2006). In addition, major facilitator superfamily (MFS) domain-containing proteins, a group of transmembrane transporters, were largely present in *F. phalaenopsidis* LS region. The MFS transporters were considered to involve in multidrug resistance. For instance, *Mycosphaerella graminicola MgMfs1* attributes tolerance to strobilurin fungicides and cercosporin (Roohparvar et al., 2007). *Colletotrichum higginsianum ChMfs1* is involved in the development of primary and secondary hyphae, conidiation, and pathogenicity on *Arabidopsis thaliana* (Liu et al., 2017). Furthermore, the predicted effectors resided on the LS region of *F. phalaenopsidis* may play important roles in virulence. For example, the gene g13479 was predicted to encode hydrophobin. A class I hydrophobin encoded by *Magnaporthe grisea MPG1* is essential in appressorium formation, conidiation, and is required for pathogenicity on rice (Talbot et al., 1996). In addition, the gene g6210 encodes a protein belonging in peptidase A4 family. *Fusarium oxysporum* f. sp. *lycopersici* peptidase FoAPY1 and aspartic protease FolAsp both contribute to virulence on tomato (Qian et al., 2022; Wang et al., 2023). This information suggests that the LS region of *F. phalaenopsidis* harbors many genes involved in the pathogenesis of *Phalaenopsis* leaf yellowing.

Previous studies have generated numerous specific primers for identification and detection of species within the FSSC. The majority of these primers are based on conserved housekeeping genes, such as *TEF1α* (Arif et al., 2012; Costa et al., 2017; Li and Hartman, 2003; Mehl and Epstein, 2007; Villarino et al., 2021) and *RPB2* (Agee and Barthet, 2021). Here, whole-genome comparison approach was employed to uncover unique gene sequences of virulence-related proteins in *F. phalaenopsidis* for specific primer design. This strategy offers several advantages. First, whole-genome information is allowed to discover unique gene sequence and non-coding sequence differentiation among intra-genus species for species-specific primer design. For instance, Vázquez-Rosas-Landa et al. (2021) compared 63 species within FSSC associated with ambrosia beetles to discover genes of unique proteins for the designation of three sets of *F. kuroshium*-specific primers. In addition, a non-conserved intergenic region obtained via comparing genome of 29 *Colletotrichum* spp. was used to design a specific primer for *C. siamense* and *C. fructicola* on strawberry (Chung et al., 2022). Second, virulence factors and effectors are often targeted for primer design due to their potential in differentiation of host range among pathogens (Lievens et al., 2009). For instance, two secreted in xylem (SIX) effector genes, *namely, SIX14* and *SIX9,* were applied to develop specific primers for identification of *F. oxysporum* f. sp. *rapae* and *F. oxysporum* f. sp. *matthiolae,* respectively (Chu et al., 2024). Finally, pathogenicity and virulence-related genes involved in prolonged pathogen–host interactions were considered to be absent in saprophytic fungi, making them suitable candidates for the detection of pathogenic fungi.

## 5 Conclusion

Currently, disease pathogenesis and pathogen dissemination of *Phalaenopsis* leaf yellowing is largely unknown, leading to a great limitation in developing disease control strategies. The genome analysis holds promise in characterization of gene functions and paves a way for elucidating potential virulence factors, thereby enhancing our knowledge of the pathogenesis. In comparative genomics, the genome structure of *F. phalaenopsidis* has been determined, and potential virulence factors were uncovered in three FCCs and the LS region. Moreover, by employing genomic comparisons, we have designed two sets of specific primers for *F. phalaenopsidis* to accelerate the identification procedures for investigating pathogen dissemination. Coupling with the stabilization of the taxonomic status of the pathogen of *Phalaenopsis* leaf yellowing, the pathogen can be identified by phylogenetic analysis or specific primers without pathogenicity assay in future.

## Data Availability

The datasets presented in this study can be found in online repositories. The names of the repository/repositories and accession number(s) can be found in the article/Supplementary material.

## Footnotes

^1^ https://github.com/nanoporetech/medaka
