# Supplementary material for: Identification and molecular detection of the pathogen of Phalaenopsis leaf yellowing through genome analysis
Source: Front Microbiol. 2024 Sep 24;15:1431813. doi: 10.3389/fmicb.2024.1431813 (PMC11472846; doi:10.3389/fmicb.2024.1431813)
Supplement: Supplementary file 6 [file Image_1.PDF]

Supplementary Materials

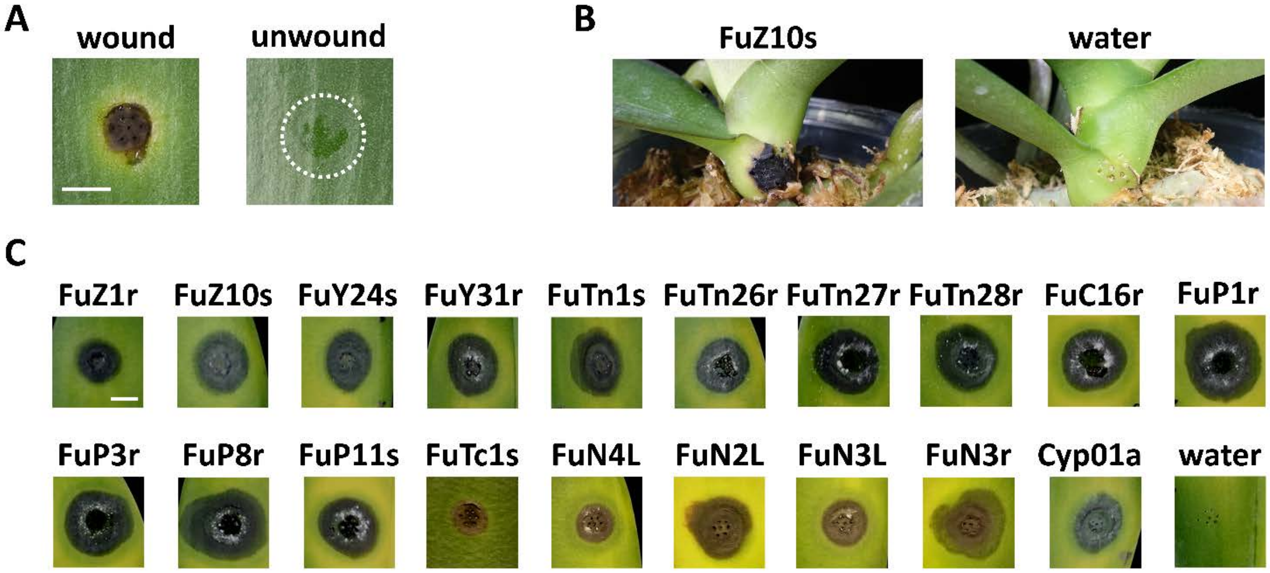

Supplementary Figure 1. Pathogenicity assay of *Fusarium phalaenopsidis* isolates on *Phalaenopsis* 'V3' leaves and stems. (A) Drop inoculation with spore suspension (10^5 spores /mL) of *F. phalaenopsidis* FuP8r on wounded and unwounded leaves. Drop inoculation on (B) leaf collar and (C) leaves. Water was used in the negative control. Scale bar: 1 cm (A).

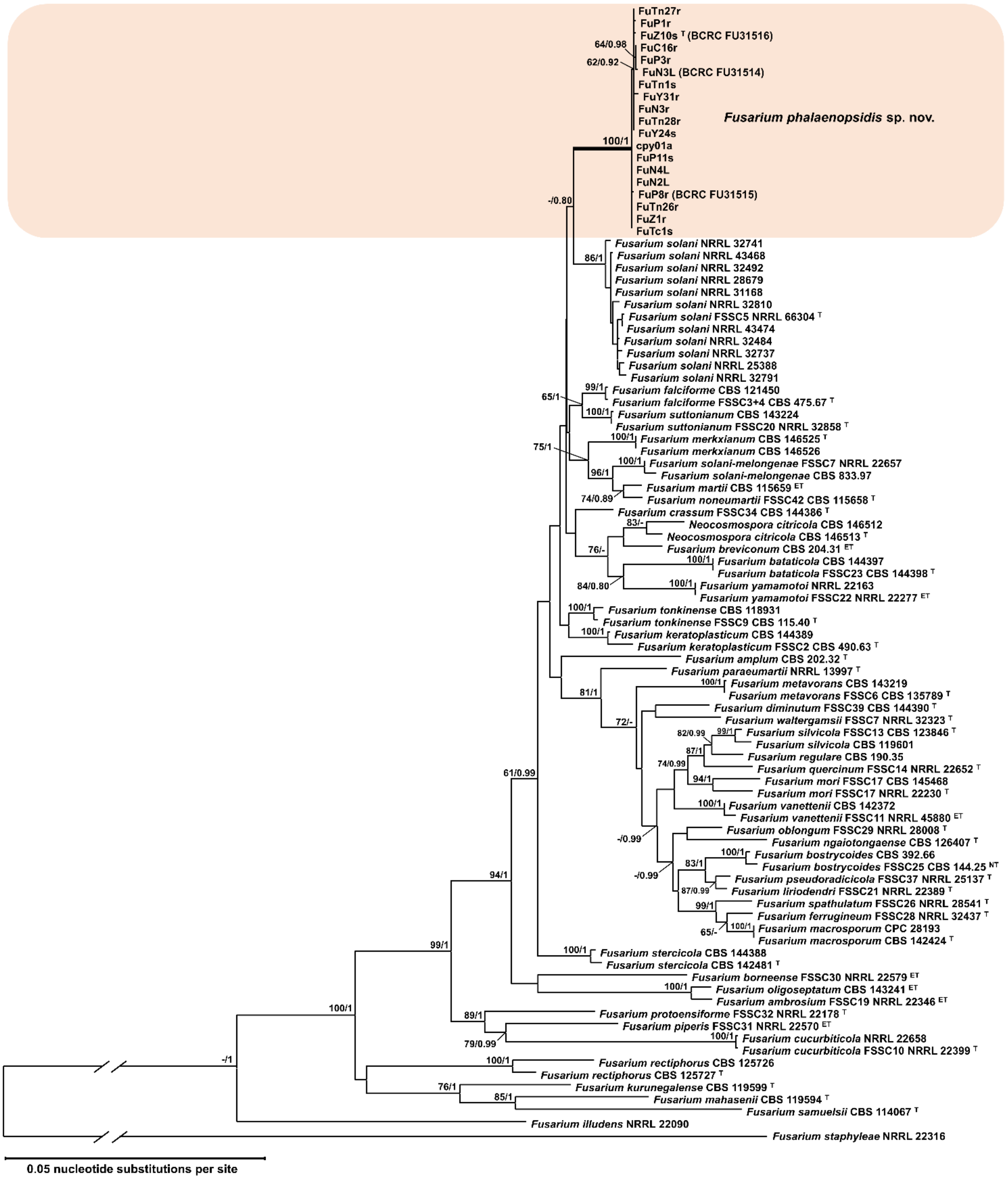
FuTn27r
FuP1r
FuZ10s T (BCRC FU31516)
64/0.98
FuC16r
62/0.92
FuP3r
FuN3L (BCRC FU31514)
FuTn1s
FuY31r
FuN3r
FuTn28r
Fusarium phalaenopsidis sp. nov.
FuY24s
100/1
cpy01a
FuP11s
FuN4L
FuN2L
FuP8r (BCRC FU31515)
-/0.80
FuTn26r
FuZ1r
FuTc1s
Fusarium solani NRRL 32741
86/1
Fusarium solani NRRL 43468
Fusarium solani NRRL 32492
Fusarium solani NRRL 28679
Fusarium solani NRRL 31168
Fusarium solani NRRL 32810
Fusarium solani FSSC5 NRRL 66304 T
Fusarium solani NRRL 43474
Fusarium solani NRRL 32484
Fusarium solani NRRL 32737
Fusarium solani NRRL 25388
Fusarium solani NRRL 32791
99/1
Fusarium falciforme CBS 121450
65/1
Fusarium falciforme FSSC3+4 CBS 475.67 T
100/1
Fusarium suttonianum CBS 143224
Fusarium suttonianum FSSC20 NRRL 32858 T
100/1
Fusarium merkxianum CBS 146525 T
75/1
Fusarium merkxianum CBS 146526
100/1
Fusarium solani-melongenae FSSC7 NRRL 22657
96/1
Fusarium solani-melongenae CBS 833.97
Fusarium martii CBS 115659 ET
74/0.89
Fusarium noneumartii FSSC42 CBS 115658 T
Fusarium crassum FSSC34 CBS 144386 T
83/-
Neocosmospora citricola CBS 146512
Neocosmospora citricola CBS 146513 T
76/-
Fusarium breviconum CBS 204.31 ET
100/1
Fusarium bataticola CBS 144397
Fusarium bataticola FSSC23 CBS 144398 T
84/0.80
100/1
Fusarium yamamotoi NRRL 22163
Fusarium yamamotoi FSSC22 NRRL 22277 ET
100/1
Fusarium tonkinense CBS 118931
Fusarium tonkinense FSSC9 CBS 115.40 T
100/1
Fusarium keratoplasticum CBS 144389
Fusarium keratoplasticum FSSC2 CBS 490.63 T
Fusarium amplum CBS 202.32 T
Fusarium paraeumartii NRRL 13997 T
81/1
100/1
Fusarium metavorans CBS 143219
Fusarium metavorans FSSC6 CBS 135789 T
Fusarium diminutum FSSC39 CBS 144390 T
72/-
Fusarium waltergamsii FSSC7 NRRL 32323 T
82/0.99
99/1
Fusarium silvicola FSSC13 CBS 123846 T
Fusarium silvicola CBS 119601
87/1
Fusarium regulare CBS 190.35
74/0.99
Fusarium quercinum FSSC14 NRRL 22652 T
94/1
Fusarium mori FSSC17 CBS 145468
61/0.99
Fusarium mori FSSC17 NRRL 22230 T
100/1
Fusarium vanettenii CBS 142372
Fusarium vanettenii FSSC11 NRRL 45880 ET
Fusarium oblongum FSSC29 NRRL 28008 T
-/0.99
Fusarium ngaiotongaense CBS 126407 T
100/1
Fusarium bostrycoides CBS 392.66
83/1
Fusarium bostrycoides FSSC25 CBS 144.25 NT
-/0.99
Fusarium pseudoradicicola FSSC37 NRRL 25137 T
94/1
87/0.99
Fusarium liriodendri FSSC21 NRRL 22389 T
99/1
Fusarium spathulatum FSSC26 NRRL 28541 T
Fusarium ferrugineum FSSC28 NRRL 32437 T
65/-
100/1
Fusarium macrosporum CPC 28193
99/1
Fusarium macrosporum CBS 142424 T
100/1
Fusarium stercicola CBS 144388
Fusarium stercicola CBS 142481 T
Fusarium borneense FSSC30 NRRL 22579 ET
100/1
Fusarium oligoseptatum CBS 143241 ET
100/1
Fusarium ambrosium FSSC19 NRRL 22346 ET
89/1
Fusarium protoensiforme FSSC32 NRRL 22178 T
Fusarium piperis FSSC31 NRRL 22570 ET
79/0.99
100/1
Fusarium cucurbiticola NRRL 22658
Fusarium cucurbiticola FSSC10 NRRL 22399 T
-/1
100/1
Fusarium rectiphorus CBS 125726
Fusarium rectiphorus CBS 125727 T
76/1
Fusarium kurunegalense CBS 119599 T
85/1
Fusarium mahasenii CBS 119594 T
Fusarium samuelsii CBS 114067 T
Fusarium illudens NRRL 22090
Fusarium staphyleae NRRL 22316
0.05 nucleotide substitutions per site

Supplementary Figure 2. Phylogenetic analysis of *Fusarium phalaenopsidis* isolates. The phylogenetic tree inferred from concatenated sequences of ITS, *TEF 1α*, *LSU* and *RPB2* was generated by the Maximum Likelihood (ML) method with the General Time Reversible model. Values at nodes indicate bootstrap values > 60 % (1000 replications) and the Bayesian posterior probability values > 0.9. The tree is rooted to *Fusarium staphyleae* NRRL 22316. T, ex-type strain. ET, ex-epitype strain.

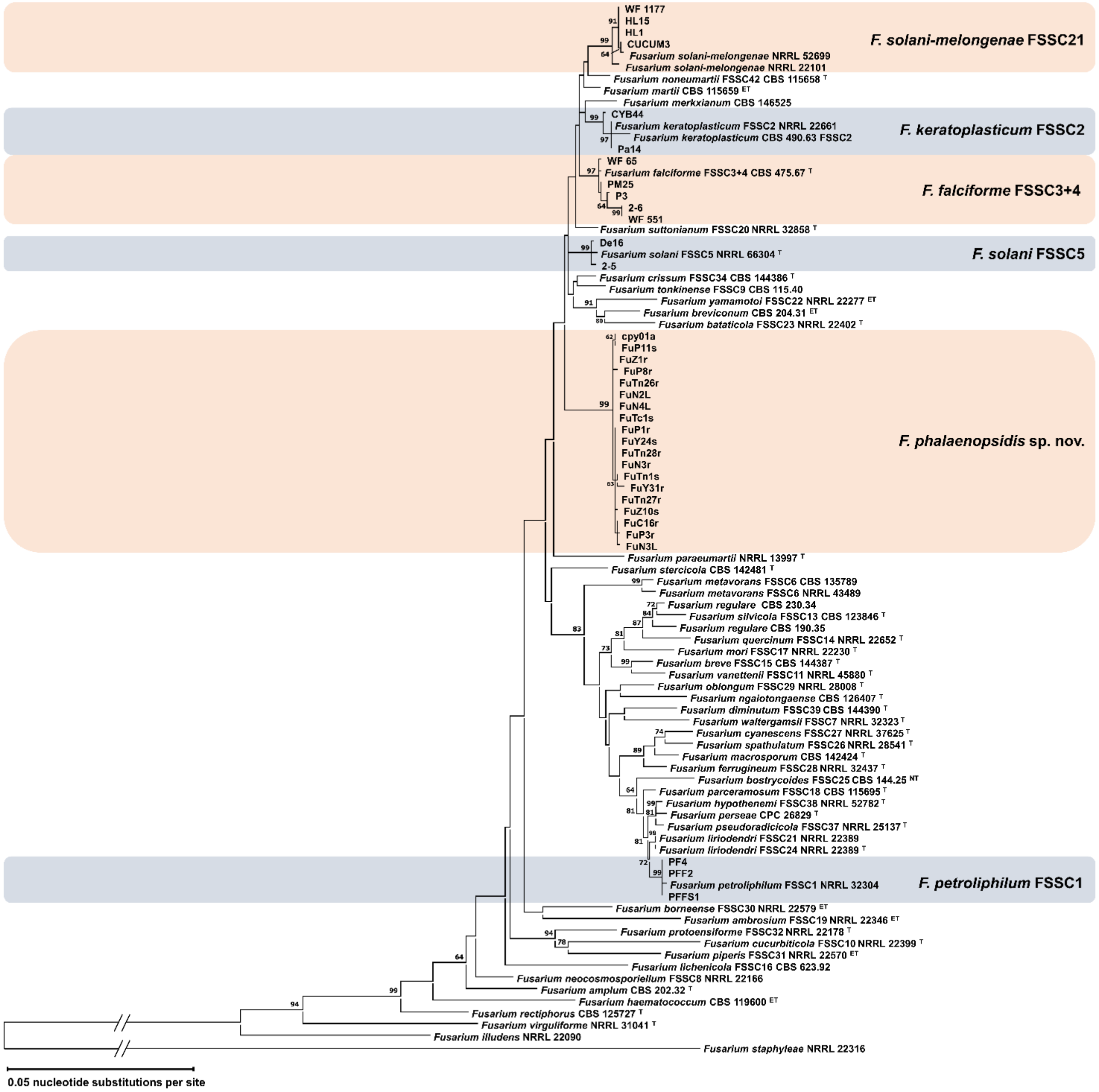

Supplementary Figure 3. Phylogenetic analysis of non-target isolates of *Fusarium solani* species complex used in this study. The phylogenetic tree inferred from concatenated sequences of *TEF1* and *RPB2* was generated by the Maximum Likelihood (ML) method with the TN93+G+I model. Values at nodes indicate bootstrap values > 60 % (1000 replications). The tree is rooted to *Fusarium staphyleae* NRRL 22316. T, ex-type strain. ET, ex-epitype strain. The symbol '// 'means abbreviation of 1 time of length based on the scale bar.

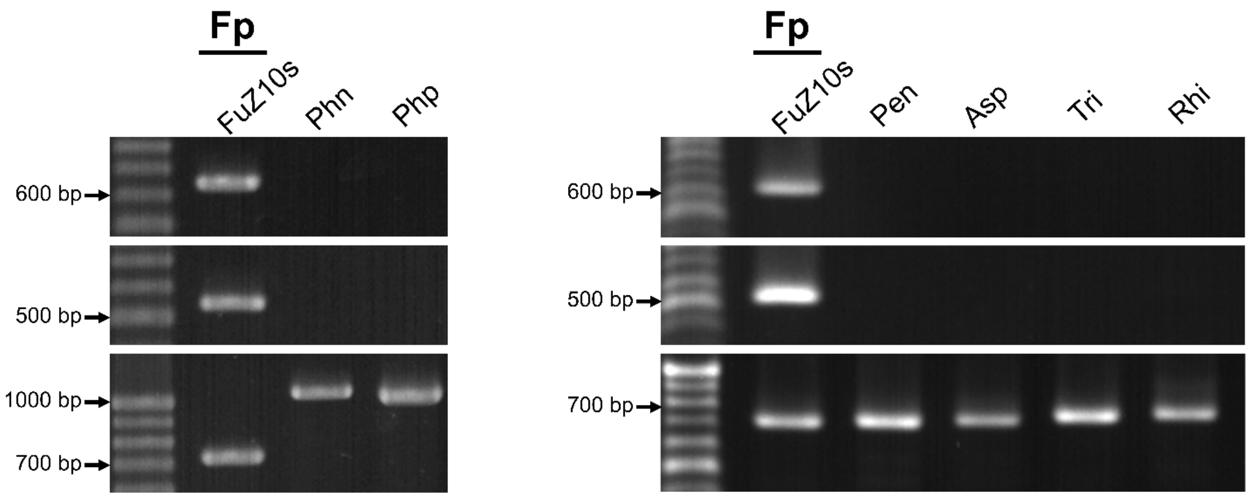

Supplementary Figure 4. Primer specificity assays on non-*Fusarium* isolates. The primer sets used in assays are FphSP1F/FphSP1R (upper panel) and FphSP6F/FphSP6R (middle panel) for detecting *Fusarium phalaenopsidis* (Fp) and V9G/ITS4 (lower panel) for verifying quality of DNA from tested isolates. Phn, *Phytophthora nicotianae*; Php, *Phytophthora palmivora*; Pen, *Penicillium* sp.; Asp, *Aspergillus* sp.; Tri, *Trichoderma* sp., Rhi: *Rhizopus* sp.

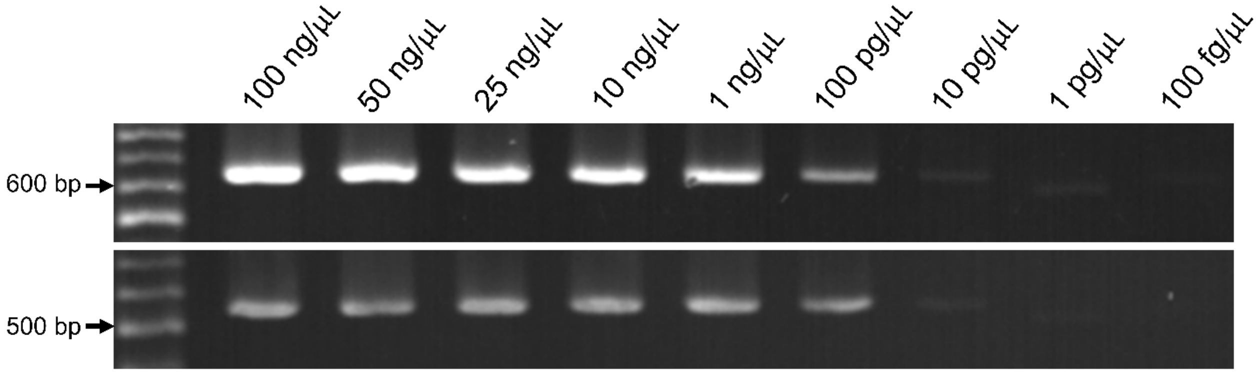

Supplementary Figure 5. Sensitivity assay of specific primers. The primers used in the upper panel and the lower panel were FphSP1F/FphSP1R and FphSP6F/FphSP6R, respectively. The DNA concentrations of *Fusarium phalaenopsidis* FuZ10s in this assay were indicated.
